# Continuity of care experienced by patients in a multi-institutional pancreatic care network: a pilot study

**DOI:** 10.1186/s12913-021-06431-2

**Published:** 2021-05-03

**Authors:** J. S. Hopstaken, D. van Dalen, B. M. van der Kolk, E. J. M. van Geenen, J. J. Hermans, E.C. Gootjes, H. J. Schers, A. M. van Dulmen, C. J. H. M. van Laarhoven, M. W. J. Stommel

**Affiliations:** 1 Department of Surgery, Radboud university medical center, Geert Grooteplein 10 (route 618), 6525 GA Nijmegen, the Netherlands; 2 Radboud Institute for Health Sciences, Radboud university medical center, Nijmegen, the Netherlands; 3 Department of Gastroenterology, Radboud university medical center, Nijmegen, the Netherlands; 4 Department of Medical Imaging, Radboud university medical center, Nijmegen, the Netherlands; 5 Department of Medical Oncology, Radboud university medical center, Nijmegen, the Netherlands; 6 Department of Primary and Community Care, Radboud university medical center, Nijmegen, the Netherlands; 7 Nivel (Netherlands institute for health services research), Utrecht, the Netherlands

**Keywords:** Pancreatic tumor, Pancreatic surgery, Continuity of care, Quality of care, Centralization, Oncology networks

## Abstract

**Background:**

Over the past decades, health care services for pancreatic surgery were reorganized. Volume norms were applied with the result that only a limited number of expert centers perform pancreatic surgery. As a result of this centralization of pancreatic surgery, the patient journey of patients with pancreatic tumors has become multi-institutional. To illustrate, patients are referred to a center of expertise for pancreatic surgery whereas other parts of pancreatic care, such as chemotherapy, take place in local hospitals. This fragmentation of health care services could affect continuity of care (COC). The aim of this study was to assess COC perceived by patients in a pancreatic care network and investigate correlations with patient-and care-related characteristics.

**Methods:**

This is a pilot study in which patients with (pre) malignant pancreatic tumors discussed in a multidisciplinary tumor board in a Dutch tertiary hospital were asked to participate. Patients were asked to fill out the Nijmegen Continuity of Care-questionnaire (NCQ) (5-point Likert scale). Additionally, their patient-and care-related data were retrieved from medical records. Correlations of NCQ score and patient-and care-related characteristics were calculated with Spearman’s correlation coefficient.

**Results:**

In total, 44 patients were included (92% response rate). Pancreatic cancer was the predominant diagnosis (32%). Forty percent received a repetition of diagnostic investigations in the tertiary hospital. Mean scores for personal continuity were 3.55 ± 0.74 for GP, 3.29 ± 0.91 for the specialist and 3.43 ± 0.65 for collaboration between GPs and specialists. Overall COC was scored with a mean 3.38 ± 0.72. No significant correlations were observed between NCQ score and certain patient-or care-related characteristics.

**Conclusion:**

Continuity of care perceived by patients with pancreatic tumors was scored as moderate. This outcome supports the need to improve continuity of care within multi-institutional pancreatic care networks.

**Supplementary Information:**

The online version contains supplementary material available at 10.1186/s12913-021-06431-2.

## Introduction

Patients suspected of pancreatic cancer go through an intensive process of multiple diagnostic investigations. If diagnosis is confirmed, they will often receive a multimodal treatment. This process usually starts with the general practitioner (GP). After referral to the hospital, several different medical professionals are involved, such as pancreatic surgeons, gastroenterologists, radiologists, medical oncologists, radiation oncologists and dieticians. In case of advanced malignant disease, palliative health care professionals are involved as well. In several European countries, such as the United Kingdom and the Netherlands, volume standards apply for pancreatic surgery [1], leading to centralization of pancreatic care in high-volume centers. The main rationale for this centralization is the significant correlation between higher hospital volume and improvement of both in-hospital mortality and long-term survival [2–4]. Evidently, these better outcomes are worth pursuing, but centralization of a part of the patient journey also carries a risk of fragmentation of care. Patients may undergo their initial diagnostic assessment in a local general hospital, have consequent or repeat diagnostic investigations and pancreatic surgery in a center of expertise, followed by adjuvant chemotherapy and follow-up in their local hospital. Additionally, during this patient journey, patients often have irregular contacts with their GPs. This multidisciplinary and multi-institutional care pathway is fragmented and may hamper continuity of care as experienced by patients suspected of pancreatic cancer.

Continuity of care (COC) is defined as the degree to which a series of healthcare events is experienced as coherent, connected and relevant to the patients’ medical requirements and personal context [5] and is associated with increased patient satisfaction, increased health-related quality of life [6, 7], decreased use of hospital services and reductions in mortality [8]. For these reasons, COC can be considered an important aspect of high-quality patient care [9, 10]. Perhaps continuity of care is even more important for pancreatic cancer patients. These patients generally render a poor prognosis and are so much dependent on different care providers that they should be able to feel assured of COC and therefore the best possible outcomes [11].

Previous studies have evaluated the experienced COC in different patient populations, such as oral cancer [12], hypertension [13], and rehabilitation [14]. However, to date no study has assessed COC in patients with (pre) malignant pancreatic tumors. This is important to assess as it may well be that the current multidisciplinary and multi-institutional pancreatic care services affect COC. In addition, such an assessment of COC may indicate areas of improvement in current pancreatic care.

The aim of this study is to assess COC experienced by patients treated in a regional pancreatic care network. A secondary aim is to investigate correlations between COC and patient-related or care-related characteristics.

## Methods

### Statement of ethics

This study was approved by the medical ethics committee (protocol number 2019–5735) of the Radboud university medical center (Radboudumc). Written informed consent was obtained for all included patients. No external incentives were provided.

### Study design

This is a pilot study with a prospective cohort of patients diagnosed with pancreatic tumors in a tertiary health center in the Netherlands. This study report is in accordance with the STROBE-guidelines (Strengthening the Reporting of Observational Studies in Epidemiology) [15].

### Setting and study population

Patients discussed in the weekly multidisciplinary tumor board meetings (MDT) of the PACON (Pancreatic Center East Netherlands) held between October 1, 2018 and December 31, 2018 were invited to participate. The PACON is part of the Radboudumc, a tertiary medical center serving six affiliated general hospitals regarding pancreatic care in the surrounding region, consisting of approximately two million inhabitants. The pancreatic care network is characterized by an anchor establishment, the Radboudumc, offering a vast array of pancreatic tumor care services, such as pancreatic surgery, and the affiliating hospitals, offering a more limited array of pancreatic tumor care services, such as systemic treatment. In order to provide appropriate care, these affiliating hospitals need to collaborate closely with the Radboudumc and refer patients if necessary. GPs can also be considered part of the network, as in the Netherlands they are the gatekeepers to secondary care. If required, they refer patients to the hospital and play a role during the entire disease trajectory.

Eligible patients with tumors of the pancreas, both (pre) malignant or benign tumors, who were discussed in the PACON between October 1, 2018 and December 31, 2018, were contacted by email between September 2019 and December 2019. Because they were referred to the PACON a year prior, they had at least a 12-month period to experience pancreatic care in the network. In case of no response, patients were sent a reminder by email and called once by a team member.

Patients aged under 18 years, those with speaking or reading difficulties in the Dutch language, and patients without a therapeutic relationship with a healthcare professional of Radboudumc regarding their pancreatic tumor, were excluded.

### Data collection

Clinical data and treatment characteristics were retrieved from the patient’s electronic medical record. These included sex, age, time of referral, referring center and discipline, type and number of diagnostic investigations in the diagnostic phase in the referring center and in the tertiary center. A repetition of diagnostics was defined as a repeat diagnostic investigation in the tertiary center within 5 months after the same diagnostic investigation. Furthermore, number of MDTs, MDT advice, type of treatment, start of treatment, number of consultations, either at the outpatient clinic or by telephone, were retrieved. Number of consultations are considered up until 1 April 2019.

### Nijmegen continuity of care questionnaire (NCQ)

The NCQ is an instrument that measures COC experienced by patients in primary and secondary care settings [16, 17]. It comprises 28 items divided into two subdomains: ‘personal continuity’ and ‘team/cross-boundary continuity’. The assessment of personal continuity involves 16 questions concerning the relationship with the patient’s health care provider, i.e. the general practitioner and the most important medical specialist, as determined by the patient. This personal continuity assesses the patient’s perception of how well the care provider knows the patient and how committed the care provider is. The second domain assesses ‘team/cross-boundary continuity’ with 12 questions, indicating the patient’s perceived COC throughout the team of primary care, the team of specialized hospital care and the cooperation between these two. Each question can be answered on a 5-point Likert scale ranging from 1 (“strongly disagree”), 2 (“disagree”), 3 (“neutral”), 4 (“agree”) to 5 (“strongly agree”), or with “?” (“I do not know”). Each subscale eventually has a mean score. NCQ-scores below 2 are interpreted as low, a mean score of 3 as moderate and mean scores of 4 or higher as high continuity of care. Additional file 1: Appendix A shows the full questionnaire. The questionnaire is considered a reliable, comprehensive instrument that measures COC as a multidimensional concept, regardless of comorbidity or care setting. It has been validated in primary care and secondary care, as well as in other languages [12, 13, 18]. To adequately assess COC, patients should have had contact with a GP or medical specialist in the previous year. The NCQ was developed to enable baseline measurements in the evaluation of interventions aiming for improved COC.

In this study, patients received a secured email providing access to the online questionnaire. After finalizing the questionnaire, patients were given the opportunity to write down additional remarks in a blank box. The NCQ-questionnaire was used in the original Dutch language and was not adjusted.

### Reliability of the NCQ-questionnaire for patients with pancreatic tumors

Since the NCQ was used in its original version, a factor analysis with content and construct validity as done by the original study [16] was deemed unnecessary. However, to indicate the robustness of the NCQ for the pancreatic tumor population, reliability was tested with the calculation of Cronbach’s alfa. Cronbach’s alfa indicates the reliability of questionnaire-items, in which alfa values > 0.8 indicate good reliability [19]. In addition, the numbers and percentages of patients with the highest and lowest score possible (ceiling and floor effects) were presented and compared to the floor and ceiling effects as observed by the original study. Floor and ceilings effects are considered to be present if > 15% of the respondents score the highest or lowest score possible [20]. With the Cronbach’s alfa and trends in ceiling and floor effects, the reliability and usefulness of this questionnaire for the pancreatic tumor population was determined.

### Statistical analysis

Data was analyzed using SPSS version 25 (SPSS Inc., Chicago, IL, USA). Descriptive data was presented as mean and standard deviation (SD) or median and range as appropriate. Categorical variables were expressed in numbers and percentages. NCQ-item scores were expressed in item means, SD and subscale scores. Patients who answered the first subscale item with ‘this subscale is not applicable to me’ and patients who answered a subscale item with ‘I do not know’ or ‘?’ were reported as missing rates. We reported the number of missing values per item and we did not compensate for missing values by imputation. A minimum of three items per subscale were required to be filled out for being considered in the analyses. Differences between GPs and medical specialists concerning mean NCQ scores were calculated using a Paired Samples T-test. Correlations between NCQ scores and patient characteristics were calculated with a Spearman’s correlation coefficient. Differences in baseline characteristics between patients with insufficient (< 3) and sufficient NCQ score (≥3) groups were calculated with a Mann-Whitney U test. *P*-values < 0.05 were considered statistically significant.

## Results

**Fig. 1:**
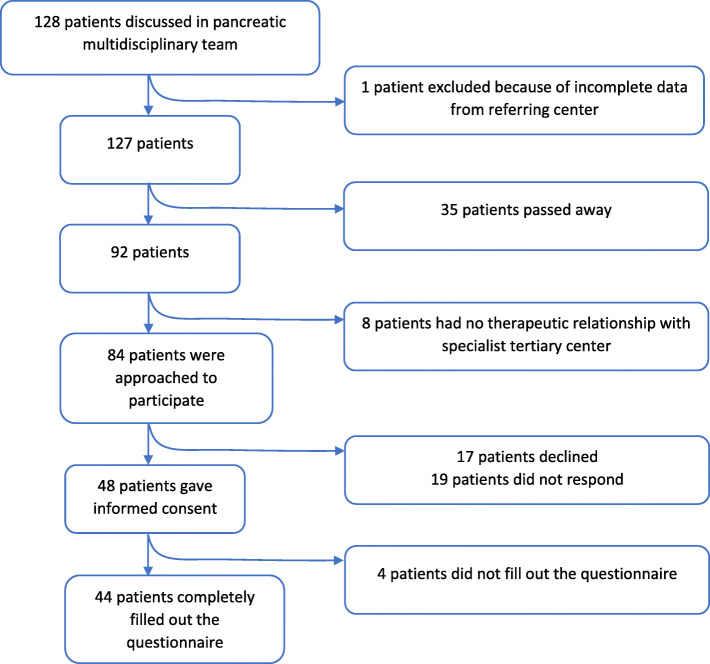
Flowchart of the total cohort and included patients

Between October 2018 and December 2018, 128 patients were discussed in the MDT of the PACON. Of these, 84 met the inclusion criteria and were approached to participate. Forty-eight patients gave informed consent, 19 patients did not respond and 17 patients declined study participation for various reasons (e.g. seriousness of disease, lack of personal benefit or because of dissatisfaction regarding their patient journey). Of the 48 patients who gave informed consent, 44 patients completed the questionnaires (92% response rate). Figure 1 depicts a flow diagram.

### Patient characteristics

Table 1 shows the baseline characteristics of the study population. The respondents had a mean age of 68 years, the majority were patients with a new diagnosis (68.2%) and male (65.9%). Pancreatic cancer was the predominant diagnosis in the cohort of NCQ respondents (31.8%), followed by the pancreatic cystic neoplasms (20.5%) and benign tumors due to chronic pancreatitis (20.5%). In the total cohort discussed in the MDT between October 2018 and December 2018 (*n* = 128) pancreatic cancer (63.3%) was the predominant diagnosis as well, followed by chronic pancreatitis (10.9%). Patients were referred to the PACON from nine different hospitals. In addition, one patient was referred directly by a GP and one patient was referred from a different department within the Radboudumc. Most patients were referred by a gastroenterologist (79.5%). A considerable proportion of the diagnostic investigations in the tertiary center was a repetition of earlier performed diagnostic investigations of the same modality in the referring hospital. For instance, of all patients receiving an abdominal CT-scan in the tertiary center, 42% had already received a prior CT-scan in the referring center. Considering the location of treatment and follow-up, the majority of patients received treatment and follow-up in the tertiary center or a combination of tertiary center and referring center.

**Table 1:** Baseline characteristics of study population (*n* = 44)

Patient Characteristic	*n* (%)^a^
Age, yrs. (mean ± sd)	67.8 ± 9.1
Male sex	29 (65.9)
Patients with new diagnosis	30 (68.2)
Diagnosis	
Pancreatic carcinoma	12 (27.3)
Metastatic pancreatic cancer	2 (4.5)
pNET	3 (6.8)
Pancreatic cystic neoplasms	9 (20.5)
Bile duct cancer/ Ampullary carcinoma	4 (9.1)
Chronic pancreatitis	9 (20.5)
Other (e.g. metastases from elsewhere)	6 (13.6)
Number of MDTs per patient (mean ± sd)	2.0 ± 1.1
Repetition of CT-scan by tertiary center (PACON)	8 (42)
Repetition of MRI-scan by tertiary center (PACON)	12 (37.5)
Repetition of endoscopy by tertiary center (PACON)	4 (25)
Time (days) between diagnosis and treatment plan by PACON^b^ (mean ± sd)	10.3 ± 15.8
Treatment advice	
Follow-up	14 (31.8)
Surgery	10 (22.7)
Neoadjuvant treatment with surgery	3 (6.8)
Surgery with adjuvant chemotherapy	6 (13.6)
Chemotherapy	1 (2.3)
Other	8 (18.2)
No treatment or no follow up indicated	2 (4.5)
Location of treatment	
Tertiary center (PACON)	25 (56.8)
Referring hospital	4 (9.1)
Combination of PACON and referring hospital	7 (15.9)
Unknown/other	8 (18.2)
Outpatients visits to specialist tertiary center (mean ± sd)	3.15 ± 2.65
Phone consultation with specialist tertiary center (mean ± sd)	2.23 ± 1.50

*Abbreviations*: *pNET* pancreatic neuroendocrine tumor, *MDT* Multidisciplinary team meeting, *PACON* Pancreatic Center East Netherlands (part of tertiary health center)

^a^unless specified otherwise in patient characteristic. ^b^Follow-up patients were excluded

### Reliability of NCQ

Floor effects were not observed. Ceiling effects were observed in three items for the GP and two items for the specialist, both in subscale 1 (Table 2). For example, the item “This care provider knows my medical history very well” was scored with 5 (1–5) by 18.2% of the patients for the GP and 22.7% of the patients for the specialist. The subscales of the NCQ appeared to have good internal consistencies for both personal continuity (Cronbach’s α = 0.91) and cross-boundary continuity (α = 0.93), as both subitems have a Cronbach’s alfa > 0.8. The Cronbach’s alfa did not increase with the removal of certain subscale items, indicating that all items were valuable to the questionnaire.

**Table 2:** Personal continuity experienced by patients in a regional pancreatic care network, concerning GP and most important specialist (ranked on a 1–5 Likert scale)

	General practitioner				Specialist			
	Mean (SD)	Missing/? (%)	Floor effect n (%)	Ceiling effect n (%)	Mean (SD)	Missing/? (%)	Floor effect n (%)	Ceiling effect n (%)
**Subscale 1: personal continuity: care provider knows me**								
a. I know this care provider very well	3.81 (0.86)	8 (18)	0 (0)	8 (18.2)	3.28	4 (9)	3 (6.8)	1 (2.3)
b. This care provider knows my medical history very well	4.03 (0.65)	8 (18)	0 (0)	8 (18.2)	3.92	7 (16)	0 (0)	10 (22.7)
c. This care provider always remembers what he/she did during my last visit(s)	3.86 (0.81)	9 (20)	0 (0)	7 (15.9)	3.81	7 (16)	1 (2.3)	8 (18.2)
d. This care provider knows my family circumstances very well	3.47 (1.06)	8 (18)	1 (2.3)	5 (11.4)	2.92	5 (11)	6 (13.6)	2 (4.5)
e. This care provider knows my daily activities very well	3.29 (0.93)	9 (20)	1 (2.3)	2 (4.5)	3.00	6 (14)	5 (11.4)	0 (0)
**Subscale 2: personal continuity: care provider shows commitment**								
f. This care provider contacts me if it is needed, I do not have to ask	3.36 (1.13)	8 (18)	0 (0)	6 (13.6)	3.38	7 (16)	2 (4.5)	4 (9.1)
g. This care provider knows very well what I believe is important in my care	3.39 (1.09)	11 (25)	2 (4.5)	4 (9.1)	3.37	6 (14)	4 (9.1)	3 (6.8)
h. This care provider keeps in contact sufficiently when I see other care providers	3.26 (0.99)	10 (23)	2 (4.5)	2 (4.5)	3.08	6 (14)	3 (6.8)	1 (2.3)
Mean score	3.55 (0.74)				3.29 (0.91)			

### The Nijmegen continuity of care questionnaire (NCQ)

In Tables 2 and 3 all mean scores are presented. The total mean NCQ score was 3.38 ± 0.72 (scale 1–5). Mean scores for personal continuity were higher for the GP than for the specialist (3.55 vs 3.29) and mean scores for cross-boundary continuity were higher for the specialist (3.66 vs 3.49), but these differences were not statistically significant. COC experienced in collaboration between GPs and specialists was ranked with 3.43 ± 0.65. Thirteen patients (29%) had a mean NCQ score below three. One of these patients had an additional remark concerning hospital care: “I have good experiences in one department, but the other department did not show commitment and did insufficiently read my medical record. For example, they did not read the treatment plan I had made with my other specialist, which led to mistakes.” Notably, more questions in subscale 3. were answered with ‘?’ or were missing compared to questions for subscale 1. and 2. Subscale 3 items concerning the collaboration within the hospital were answered by approximately 65% of all respondents whereas items concerning collaboration within primary care were answered by 46% of all respondents.

**Table 3:** Continuity of care experienced by patients in a regional pancreatic care network in collaboration between care providers (ranked on a 1–5 Likert scale

	Within primary care				Within the hospital				Between general practitioner and specialist			
	Mean (SD)	Missing/? (%)	Floor effect n (%)	Ceiling effect n (%)	Mean (SD)	Missing/? (%)	Floor effect n (%)	Ceiling effect n (%)	Mean (SD)	Missing/? (%)	Floor effect n (%)	Ceiling effect n (%)
**Subscale 3: Cross-boundary continuity**												
1. These care providers transfer information very well to each other	3.45 (0.86)	22 (50)	1 (2.3)	1 (2.3)	3.76 (0.51)	15 (34)	0 (0)	1 (2.3)	3.64 (0.73)	16 (36)	0 (0)	3 (6.8)
2. These care providers work together very well	3.60 (0.68)	24 (55)	0 (0)	1 (2.3)	3.71 (0.60)	16 (36)	0 (0)	1 (2.3)	3.44 (0.64)	17 (38)	0 (0)	0 (0)
3. The care of these care providers is very well-connected	3.50 (0.61)	24 (55)	0 (0)	0 (0)	3.76 (0.69)	15 (34)	0 (0)	3 (6.8)	3.41 (0.78)	17 (38)	0 (0)	1 (2.3)
4. These care providers always know very well from each other what they do	3.32 (0.82)	25 (57)	1 (2.3)	0 (0)	3.43 (0.84)	16 (36)	0 (0)	2 (4.5)	3.15 (0.78)	18 (41)	0 (0)	1 (2.3)
Mean score	3.49 (0.68)				3.66 (0.58)				3.43 (0.65)			

### COC and correlated parameters

There were no significant correlations observed between mean NCQ scores and patient- and care-related characteristics such as diagnosis, age, number of MDT meetings, outpatient visits or the repetition of diagnostics. Additionally, no significant differences were observed between patients with an insufficient or sufficient NCQ scores (< 3 or ≥ 3) concerning these characteristics.

## Discussion

This study assessed the continuity of care experienced by patients in a regional pancreatic care network and investigated possible correlations between COC and patient- and care-related characteristics.

Overall COC was scored with a mean 3.38 ± 0.72, which indicates that patients with pancreatic tumors in our pancreatic care network perceive COC as moderate. Mean score for personal continuity for the GP and the specialist were 3.55 ± 0.74 and 3.29 ± 0.91. Mean scores for COC in collaboration within primary care, within the hospitals and between these institutes were 3.49 ± 0.68, 3.66 ± 058 and 3.43 ± 0.65. No significant differences were observed in total scores between GPs and specialists and no significant correlations were observed between NCQ score and certain patient-related or care-related characteristics.

Comparing these results with the study by Uijen et al. (2011), the authors who developed the NCQ, several similarities and differences can be seen [16]. Similarly to our findings, as we observed higher missing rates for team/cross boundary continuity (35–38%) compared to personal continuity, Uijen et al. also describe high missing rates for the questionnaire items concerning team/cross boundary continuity (25–26%). These findings suggest that patients are more hesitant to answer this type of questions because they are not aware or informed about the collaboration within or between these care providers. However, in contrast with the Uijen et al., our study reports higher missing rates for the items concerning collaboration within primary care than for the collaboration within hospital care. This could suggest that patients included in this study perceived items concerning the collaboration within primary care to a lesser extent applicable to them. A possible explanation for this is that the GP was less involved in their care process compared to the patient population studied by Uijen et al., which consisted of patients with hypertension, diabetes mellitus type 2 and COPD. Considering floor and ceiling effects, it is noteworthy that a larger proportion of patients reported high scores for personal continuity of the specialist compared to the scores described in the original article. For instance, “This care provider always remembers what he/she did during my last visit” was scored the highest possible score by 18% of this study population, while Uijen et al. report this to be 5%. This difference suggests that personal COC provided by the specialist was better evaluated by pancreatic tumor patients than the patient population included in Uijen et al. This difference is, however, quite conceivable considering pancreatic tumors are rare for a GP and the diagnostic and treatment process, in contrast to hypertension, diabetes mellitus type 2 and COPD, generally takes place in the hospital. Other differences in floor and ceiling effects between these studies were not observed. Considering the absence of large floor and ceiling effects and the high internal consistence observed in this study (Cronbach’s alpha > 0.9), we have shown that the NCQ is a reliable and useful tool to measure COC experienced by patients in a regional pancreatic care network.

This study has several limitations. Firstly, this study has a low sample size. This is partially due to the explorative character of the study. We intended to quantitatively measure how COC was experienced by patients in our network as we learned from some patient interviews that COC was experienced as suboptimal. In order to assess this, we aimed to include approximately 50 patients from our total cohort to fill out the NCQ. We considered this to be an adequate number of patients for gaining insight in the experienced COC, and to test whether the NCQ was a reliable tool to assess COC in patients with pancreatic tumors. Another reason for the low sample size, is that a substantial number of patients in our cohort died over the follow-up period and became ineligible for study participation. A one-year follow-up period was required for COC to be adequately evaluated. Because of this low sample size, the lack of observed correlations between NCQ scores and certain patient characteristics should be interpreted with caution. Especially since other studies with larger sample sizes have described correlations between continuity of care and patient characteristics such as sex, diagnosis or number of outpatient visits [12, 13, 21]. Therefore, there is the possibility a type II error occurred. To circumvent the problem of a low sample size that may occur when patient experiences need to be collected as a measure of COC, several studies have suggested to use medical records or national insurance data to derive insight in COC [22–24]. From these data certain parameters, such as the total number of providers, total number of patient visits or the number of handoffs of information, can be derived. Subsequently, these parameters may serve in a COC index, such as the Usual Provider of Care, the Bice-Boxerman Continuity of Care Index, Sequential Continuity Index or the Herfindahl-Hirschman index [22, 25–27]. Though this would indeed not require participation of patients or exhaustive data collection, these quantitatively derived indices have restrictions. They only measure one component of COC and some, especially those indices focusing on the proportion of visits where the patient saw the same care provider, do not take into account aspects such as a change of GP due to intentional reasons or multiple care providers due to multimorbidity [28]. It seems more sensible to determine COC by measuring the experience of those that actually receive care – the patients. Therefore, we believe that establishing the patients’ perspective on COC is most appropriate for establishing COC. Using a patient-centered measure of COC could also be perceived as a strength. In addition to the low sample size, a second limitation concerns the representativeness of the study. Pancreatic cancer was the predominant diagnosis in both cohorts, however, in the total cohort discussed in the MDT between October 2018 and December 2018 twice as many patients had pancreatic cancer (63%) as in the cohort of NCQ respondents (32%). This difference can be partially attributed to the deaths of patients with pancreatic cancer after 1 year. The underrepresentation of patients with pancreatic cancer makes the respondents cohort less representative for the total patient population with pancreatic tumors. On the other hand, the group of respondents is a true representation of the population that is alive 1 year after discussion in an MDT. A third limitation concerns non-response bias. Non-response bias plays a role as 20% of the approached patients declined participation because of several reasons, of which one was dissatisfaction with current care. The valuable input from this patient category is not reflected in our study results.

A strength of this study is that it is the first to describe COC as perceived by patients with pancreatic tumors in a pancreatic care network. Additionally, we have shown that the NCQ is a reliable questionnaire for this patient population. As personal and cross-boundary continuity were evaluated both for primary and hospital care, a comprehensive evaluation of COC for pancreatic tumor patients is obtained, revealing new information in the domain of overall quality of care for this patient population. Considering very limited studies have focused on quality of care and health services research for patients with pancreatic tumors [29], this study makes a valuable contribution.

Because COC is experienced as only moderate in patients with suspected pancreatic cancer, improvement of COC deserves attention. The extremely low five-year survival rates of pancreatic cancer patients [11], emphasizes the relevance of quality of life and quality of care for this patient population. The results of this study support the urge for coherent and better connected care. In addition, the complexity of managing pancreatic tumor patients in a multi-institutional network is illustrated in this study. Almost 20% the respondents received treatment concerning their pancreatic tumor in both the general hospital and the tertiary center simultaneously. Two in five patients undergoing a diagnostic investigation in the tertiary center, such as endoscopy, a CT or MRI-scan, had already undergone this investigation in the referring hospital. Reasons for repetition were not monitored but could consists of an inadequate primary scan (i.e. wrong protocol) or loss of actuality due to delay. We suspect that if collaboration within this multi-institutional network were to be improved, unnecessary, invasive and costly repeat-diagnostic procedures could be avoided. However, studies are needed to test this hypothesis.

In literature, several interventions are described to improve COC experienced by patients with multiple care providers. Some suggest the MDT is best able to coordinate care and ensure continuity of patient care and that the involvement of a GP in the MDT, for instance by means of video call, may lead to better coordination and continuity of care [30, 31]. Others propose joint consultations with GPs, oncologists and the patient [32]. Empirical studies are momentarily lacking but the effect of these joint consultations on continuity of care is currently being studied in a Danish RCT [33]. Another possible intervention could be found in the direction of eHealth and digital care platforms [34]. Such a platform may enable care providers from multiple institutes to exchange important patient-related information, offer patients e-consultations and allow patients easy access in viewing their current treatment trajectory, appointments, and diagnostic results. Though most studies involving these platforms have a short follow-up and low sample size, the preliminary results are promising in terms of feasibility and acceptability [34]. It seems reasonable that these platforms may bridge the gaps between primary, secondary and tertiary health care and aid in making the health care services for patients with pancreatic tumors more streamlined, continuous and of high-quality. Future studies are necessary to study this hypothesis.

## Conclusion

Continuity of care perceived by patients with pancreatic tumors in our multi-institutional network was perceived as moderate. No correlations between patient- or care-related characteristics and continuity of care were observed. The moderate outcome of the perceived COC underlines the necessity to improve current COC experienced during the patient journey by for instance improving health care services. We advocate for investments by health care providers involved in the treatment of patients with pancreatic tumors to investigate possible means to improve current COC provided in a multi-institutional network.

## Supplementary Information

**Additional file 1: Appendix A.** The Nijmegen Continuity of Care Questionnaire (NCQ).

## Data Availability

In order to protect the privacy of the patients that are included in this study, data are not made available. Please contact the corresponding author (martijn.stommel@radboudumc.nl) in case of inquiries.

## Abbreviations

COC: Continuity of care
CT: Computed tomography
GP: General practitioner
MDT: Multidisciplinary team meeting
MRI: Magnetic resonance imaging
NCQ: Nijmegen Continuity of Care Questionnaire
PACON: Pancreatic Center East Netherlands (part of tertiary health center)
RCT: Randomised controlled trial
