# Supplementary material for: Continuity of care experienced by patients in a multi-institutional pancreatic care network: a pilot study
Source: BMC Health Serv Res. 2021 May 3;21:416. doi: 10.1186/s12913-021-06431-2 (PMC8094517; doi:10.1186/s12913-021-06431-2)
Supplement: Supplementary file 1 — Additional file 1: Appendix A. The Nijmegen Continuity of Care Questionnaire (NCQ). [file 12913_2021_6431_MOESM1_ESM.docx]

Supplementary material
**Appendix A:** The Nijmegen Continuity of Care Questionnaire (NCQ)

| **1. The following statements are about your (own) general practitioner (Personal Continuity)** If you did not contact a general practitioner in the last year, please go on to the next section. | | | | | | | | | | | | | | | | | | | | | | | | | | | | | | | | | |
| --- | --- | --- | --- | --- | --- | --- | --- | --- | --- | --- | --- | --- | --- | --- | --- | --- | --- | --- | --- | --- | --- | --- | --- | --- | --- | --- | --- | --- | --- | --- | --- | --- | --- |
|  | (1) Strongly disagree | | | | | (2) Disagree | | | | | (3)  Neutral | | | | | (4)  Agree | | | | | (5)  Strongly agree | | | | | ? | | | | |  |  |  |
| a. I know my general practitioner very well. | O | | | | | O | | | | | O | | | | | O | | | | | O | | | | | O | | | | |  |  |  |
| b. My general practitioner knows my medical history very well. | O | | | | | O | | | | | O | | | | | O | | | | | O | | | | | O | | | | |  |  |  |
| c. My general practitioner always remembers what he/she did previously | O | | | | | O | | | | | O | | | | | O | | | | | O | | | | | O | | | | |  |  |  |
| d. My general practitioner knows my family circumstances very well. | O | | | | | O | | | | | O | | | | | O | | | | | O | | | | | O | | | | |  |  |  |
| e. My general practitioner knows my daily activities very well. | O | | | | | O | | | | | O | | | | | O | | | | | O | | | | | O | | | | |  |  |  |
| f. My general practitioner contacts me if it is needed, I do not have to ask. | O | | | | | O | | | | | O | | | | | O | | | | | O | | | | | O | | | | |  |  |  |
| g. My general practitioner knows very well what I believe is important in my care. | O | | | | | O | | | | | O | | | | | O | | | | | O | | | | | O | | | | |  |  |  |
| h. My general practitioner keeps in contact sufficiently when I see other care providers | O | | | | | O | | | | | O | | | | | O | | | | | O | | | | | O | | | | |  |  |  |
| **2. The following are about the cooperation between care providers in general practice (e.g. between general practitioner and nurse practitioner or between several general practitioners) (Cross-boundary continuity)** If this section does not apply to you, please go on to the next section. | | | | | | | | | | | | | | | | | | | | | | | | | | | | | | | | | |
|  | (1) Strongly disagree | | | | (2) Disagree | | | | | (3)  Neutral | | | | | (4)  Agree | | | | | (5)  Strongly agree | | | | | ? | | | | |  |  |  |  |
| a. These care providers transfer information very well to each other | O | | | | O | | | | | O | | | | | O | | | | | O | | | | | O | | | | |  |  |  |  |
| b. These care providers work together very well | O | | | | O | | | | | O | | | | | O | | | | | O | | | | | O | | | | |  |  |  |  |
| c. The care of these care providers is very well connected | O | | | | O | | | | | O | | | | | O | | | | | O | | | | | O | | | | |  |  |  |  |
| d. These care providers always know very well from each other what they do | O | | | | O | | | | | O | | | | | O | | | | | O | | | | | O | | | | |  |  |  |  |
| **3. The following statements are about your (most important) specialist (Personal continuity)** If you did not contact a specialist in the last year, please go on to the next section. | | | | | | | | | | | | | | | | | | | | | | | | | | | | | | | | |  |
|  | (1) Strongly disagree | | | (2) Disagree | | | | | (3)  Neutral | | | | | (4)  Agree | | | | | (5)  Strongly agree | | | | | ? | | | | |  |  |  |  |  |
| a. I know this care provider very well. | O | | | O | | | | | O | | | | | O | | | | | O | | | | | O | | | | |  |  |  |  |  |
| b. This care provider knows my medical history very well. | O | | | O | | | | | O | | | | | O | | | | | O | | | | | O | | | | |  |  |  |  |  |
| c. This care provider always remembers what he/she did previously | O | | | O | | | | | O | | | | | O | | | | | O | | | | | O | | | | |  |  |  |  |  |
| d. This care provider knows my family circumstances very well. | O | | | O | | | | | O | | | | | O | | | | | O | | | | | O | | | | |  |  |  |  |  |
| e. This care provider knows my daily activities very well. | O | | | O | | | | | O | | | | | O | | | | | O | | | | | O | | | | |  |  |  |  |  |
| f. This care provider contacts me if it is needed, I do not have to ask. | O | | | O | | | | | O | | | | | O | | | | | O | | | | | O | | | | |  |  |  |  |  |
| g. This care provider knows very well what I believe is important in my care. | O | | | O | | | | | O | | | | | O | | | | | O | | | | | O | | | | |  |  |  |  |  |
| h. This care provider keeps in contact sufficiently when I see other care providers | O | | | O | | | | | O | | | | | O | | | | | O | | | | | O | | | | |  |  |  |  |  |
| **4. The following statements are about the cooperation between care providers in hospital (e.g. between several specialists or between specialist and nurse) (Cross-boundary continuity)** If this section does not apply to you, please go on to the next section. | | | | | | | | | | | | | | | | | | | | | | | | | | | | | | | | |  |
|  | (1) Strongly disagree | | (2) Disagree | | | | | (3)  Neutral | | | | | (4)  Agree | | | | | (5)  Strongly agree | | | | | ? | | | | |  |  |  |  |  |  |
| a. These care providers transfer information very well to each other | O | | O | | | | | O | | | | | O | | | | | O | | | | | O | | | | |  |  |  |  |  |  |
| b. These care providers work together very well | O | | O | | | | | O | | | | | O | | | | | O | | | | | O | | | | |  |  |  |  |  |  |
| c. The care of these care providers is very well connected | O | | O | | | | | O | | | | | O | | | | | O | | | | | O | | | | |  |  |  |  |  |  |
| d. These care providers always know very well from each other what they do | O | | O | | | | | O | | | | | O | | | | | O | | | | | O | | | | |  |  |  |  |  |  |
| **5. The following statements are about the cooperation between your general practitioner and your specialist. (Cross-boundary continuity)** If this section does not apply to you, than you finished the questionnaire. | | | | | | | | | | | | | | | | | | | | | | | | | | | | | | | |  |  |
|  | (1) Strongly disagree | (2) Disagree | | | | | (3)  Neutral | | | | | (4)  Agree | | | | | (5)  Strongly agree | | | | | ? | | | | |  |  |  |  |  |  |  |
| a. These care providers transfer information very well to each other | O | O | | | | | O | | | | | O | | | | | O | | | | | O | | | | |  |  |  |  |  |  |  |
| b. These care providers work together very well | O | O | | | | | O | | | | | O | | | | | O | | | | | O | | | | |  |  |  |  |  |  |  |
| c. The care of these care providers is very well connected | O | O | | | | | O | | | | | O | | | | | O | | | | | O | | | | |  |  |  |  |  |  |  |
| d. These care providers always know very well from each other what they do | O | O | | | | | O | | | | | O | | | | | O | | | | | O | | | | |  |  |  |  |  |  |  |
